# New York College of Veterinary Surgeons and School of Comparative Medicine

**Published:** 1890-04

**Authors:** 


### VETERINARY COLLEGE NOTES.

*New York College of Veterinary Surgeons and School of Comparative Medicine.*—The thirty-third annual commencement of the New York College of Veterinary Surgeons and School of Comparative Medicine was held in Chickering Hall New York, on Friday, March 7th. President W. T. White, M.D., conferred degrees upon the following nineteen gentlemen: Samuel S. Brooks, Paterson, N. J.; George Samuel Fuller, Fremont, Ohio; Sherman William Mount, Milwaukee, Wis.; Emil Alexander Mayer, New York; Peter Archibald Davison, Asbury Park, N. J.; John Joseph Fox, New York; Dennis Doyle, Freehold, N. J.; William Henry Valway, Vergennes, Vermont; Alfred Emil Luks, Ph.G., Vineland, N. J.; Josiah Winants Provoost, New York; Conrad William Braeutigam, Ph.G., Brooklyn, E. D.; Edward N. Farrell, St. Louis, Mo.; Chas. Reccord, North Raynham, Mass.; Edward Frederick Koch, Brooklyn, E. D.; William James McKinney, Londonderry, Ire.; Arthur Gilmore Carter, New York City; Thomas F. Fallon, New York City; Robert Keenan, Long Island; Moses E. Critcherson, Rhode Island.

An address was made by the Rev. William H. Lewis. Samuel S. Brooks, V.S., of the graduating class, delivered the valedictory. The class officers were: President, Arthur C. Carter; vice-president, John J. Fox; secretary, Alfred F. Luks; treasurer, George Fuller.

